# External quality assessment on the use of malaria rapid diagnostic tests in a non-endemic setting

**DOI:** 10.1186/1475-2875-9-359

**Published:** 2010-12-13

**Authors:** Philippe Gillet, Pierre Mukadi, Kris Vernelen, Marjan Van Esbroeck, Jean-Jacques Muyembe, Cathrien Bruggeman, Jan Jacobs

**Affiliations:** 1 Department of Clinical Sciences, Institute of Tropical Medicine (ITM), Antwerp, Belgium; 2 Institut National de Recherche Biomédicale (INRB), Kinshasa, Democratic Republic of the Congo; 3 Institute of Public Health (IPH), Brussels, Belgium; 4 Department of Medical Microbiology, School for Public Health and Primary Care (CAPHRI), Maastricht University Medical Centre, Maastricht, The Netherlands

## Abstract

**Background:**

Malaria rapid diagnostic tests (RDTs) are increasingly used as a tool for the diagnosis of malaria, both in endemic and in non-endemic settings. The present study reports the results of an external quality assessment (EQA) session on RDTs in a non-endemic setting.

**Methods:**

After validation of antigen stability during shipment at room temperature, three clinical samples and a questionnaire were sent to clinical laboratories in Belgium and the Grand Duchy of Luxembourg using malaria RDTs. Participants were asked to report the results of the RDTs as observations (visibility of the RDT control and test lines) and interpretations (report as formulated to the clinician). In addition, participants were invited to fill in a questionnaire on the place of RDTs in the diagnostic strategy of malaria.

**Results:**

A total of 128/133 (96.2%) of clinical laboratories using RDTs participated. Six three-band and one four-band RDT brands were used. Analytical errors were rare and included (i) not recognizing invalid RDT results (1.6%) and (ii) missing the diagnosis of *Plasmodium falciparum *(0.8%). Minor errors were related to RDT test result interpretation and included (i) reporting "RDT positive" without species identification in the case of *P. falciparum *and non-*falciparum *species (16.9% and 6.5% respectively) and (ii) adding incorrect comments to the report (3.2%). Some of these errors were related to incorrect RDT package insert instructions such as (i) not reporting the possibility of mixed species infection in the case of *P. falciparum *and *Plasmodium vivax *(35.5% and 18.5% respectively) and (ii) the interpretation of *P. vivax *instead of non-falciparum species at the presence of a pan-species antigen line (4.0%). According to the questionnaire, 48.8% of participants processed ≤20 requests for malaria diagnosis in 2009. During opening hours, 93.6% of 125 participants used RDTs as an adjunct to microscopy but outside opening hours, nearly one third of 113 participants relied on RDTs as the primary (4.4%) or the single tool (25.7%) for malaria diagnosis.

**Conclusion:**

In this non-endemic setting, errors in RDT performance were mainly related to RDT test line interpretations, partly due to incorrect package insert instructions. The reliance on RDTs as the primary or the single tool for the diagnosis of malaria outside opening hours is of concern and should be avoided.

## Background

Malaria rapid diagnostic tests (RDTs) are increasingly used as a diagnostic tool in both malaria endemic and non-endemic settings. RDTs detect *Plasmodium *parasites in blood by antibody-antigen reactions on a nitrocellulose strip, which become visible as cherry-red lines. Different formats exist: two-band RDTs are mostly designed to detect *Plasmodium falciparum*. They display a control line and a test line, which targets either histidine-rich protein-2 (HRP-2) or *P. falciparum*-specific parasite lactate dehydrogenase (Pf-pLDH). Three- and four-band RDTs display a control line and two or three test lines, one targeting a *P. falciparum *specific antigen, a second line targeting antigens common to the four species, such as pan-*Plasmodium*-specific lactate parasite dehydrogenase (pan-pLDH) or aldolase, and in case of the four band RDTs, a third line which targets *Plasmodium vivax*-specific pLDH (Pv-pLDH).

The use of RDTs is rapidly expanding: in 2007, more than 70,000,000 tests were performed and more than 80 brands were world-wide marketed [1]. In malaria-endemic regions, RDTs are currently rolled out by national malaria control programs as a tool for parasite based diagnosis [1]. In non-endemic settings, where microscopic expertise is lacking due to low incidence, RDTs have been reported to perform accurately and even better as compared to microscopy [2,3]. Despite their robust design and their ease of use, RDTs are not fail-proof and quality problems have been identified at the level of production, transport and storage as well as at the level of the end-user's performance [4-10]. Initiatives for quality assurance along the route from design to end-user have been inspired by the World Health Organization (WHO). At the level of product performance there have been the two evaluation rounds of RDTs by WHO/Foundation for Innovative New Diagnostics (FIND) [11,12]. At the level of production and distribution, WHO has set up a comprehensive quality control strategy for production, transport and product control in national reference laboratories as well as a lot testing program [13,14]. With regard to the end-user's performance, there has been the redaction of generic job aids [15,16] and the development of stable positive controls [17]. Till now, external quality assessment (EQA) sessions on the use of RDTs have not been organized. A national survey in the UK has, however, highlighted the need for such EQA sessions from the part of clinical laboratories [18]. In light of these reasons, the present EQA was organized.

## Methods

### Participants, samples and questionnaire

The present EQA session was organized among clinical laboratories in Belgium and the Grand Duchy of Luxembourg which had declared to be interested in EQA sessions on malaria rapid diagnostic tests when subscribing to the EQA session "Blood Parasites" or "Microbiology" organized by the Institute of Public Health, Brussels, Belgium. In a survey afterwards, the non-subscribing laboratories were addressed to ask whether they use RDTs or not in the diagnosis of malaria.

The EQA panel consisted of three samples of EDTA-anticoagulated blood: one sample with *P. falciparum*, another with no evidence of *Plasmodium *and a third one with *P. vivax *(Table 1). They were obtained from patients suspected of malaria presenting at the Institute of Tropical Medicine (ITM). After initial analysis and diagnosis, samples were stored at 4°C for a maximum of 48 hours and subsequently aliquoted in 150 μl-fractions which were stored at -70°C. Total durations of storage at -70°C till EQA shipment were 612, 249 and 240 days for samples 1, 2 and 3 respectively.

**Table 1:** Clinical information and parasite density of the embedded samples of the EQA session.

Sample number	History	Species, parasite density
1	Pregnant woman, Nigeria.	*P. falciparum*, 53,024/μl = 1%
2	NGO volunteer, Burkina Faso, treated for malaria 4 weeks ago.	No *Plasmodium *detected.
3	Traveler: Democratic Republic of the Congo, Haiti.	*P. vivax*, 3,251/μl = 0.06%

Diagnosis of malaria, species identification and determination of parasite density were done by microscopy and confirmed by PCR. According to standard practice at ITM, thick and thin blood films were stained with Giemsa (pH 8.0) and examined by light microscopy using a × 500 magnification [19]. Parasite densities were estimated by counting asexual parasites against 200 white blood cells (WBC) in thick blood films and converting this number to parasites/μl using the actual WBC count [8]. Parasite densities are further in this text expressed as counts (of asexual parasites)/μl (of whole blood), with 50,000 red blood cells/μl set as 1% of red blood cells [8]. Species identification was confirmed by *Plasmodium*-specific PCR [20].

In addition to the samples, a questionnaire on the performance of RDTs in clinical laboratories was prepared. This questionnaire was based on a previous survey performed in the UK [18] and addressed issues of frequency of requests of malaria diagnosis, ease of use and the place of RDTs in the diagnostic strategy.

### Validation of shipment and questionnaire

For validation of the shipment at room temperature (in particular the antigen stability), a try-out session was performed among the members of the IPH referee committee, which consists of a panel of laboratory professionals in charge of piloting EQA sessions. Aliquots of the samples at ITM were retrieved, allowed to thaw and processed at room temperature. The samples were tested against a panel of RDTs available in Belgium, as listed in Table 2. Two of these brands were not available at the time of try-out session (Cypress and Ultimed); they were performed at the moment of the formal EQA session. Aliquots were packaged according to the UN 3373 recommendations and transported the same day by car to a regular IPH referee committee meeting during which they were distributed: each member (n = 9) received two packages, one for analysis by his/her laboratory and another to be sent back by regular post mail to ITM. The latter package contained a temperature logger (Escort data loggers^®^, Buchanan, Virginia United States), making it possible to monitor the temperature during transport and shipment. Upon arrival at ITM, the returned packages with the samples were stored at 4°C for a maximum of 48 hours and RDT testing was repeated. The test line results observed upon testing these returned aliquots were compared to those that had been obtained in fresh samples.

**Table 2:** Overview of malaria RDT brands used by the participants (n = 128).

Manufacturer	Malaria RDT	Format	Target antigens	Numbers of participants (%)
Inverness Medical Binax, Inc., Scarborough, Maine, USA	BinaxNOW^® ^Malaria Test	Card box Three-band	HRP-2 + Aldolase	54 (42.1)
All Diag, Strasbourg, France	Palutop+4^®^	Cassette Four-band	HRP-2 + Pv-pLDH + pan-pLDH	26 (20.3)
DiaMed AG, Cressier s/Morat Switzerland	OptiMal-IT	Hybrid dipstick Three-band	Pf-pLDH + pan-pLDH	23 (18.0)
Access Bio Inc, New Jersey, USA	CareStart™ Malaria pLDH/HRP2 Combo test	Cassette Three-band	HRP-2 + pan-pLDH	12 (9.4)
Standard Diagnostics Inc, Hagal-Dong, Korea	SD Bioline Malaria Ag Pf/Pan FK 60	Cassette Three-band	HRP-2 + pan-pLDH	11 (8.6)
Ultimed Ahrensburg, Germany	Malaria (*P. falciparum*/pan) Test	Cassette Three-band	HRP-2 + pan-pLDH	1 (0.8)
Cypress Diagnostics, Leuven, Belgium	Malaria Total Quick Test	Cassette Three-band	HRP-2 + pan-pLDH	1 (0.8)

The underlined names represent the shortened names used in the text to refer to the different RDT brands.

All RDTs were performed according to the instructions of the manufacturer, except that the transfer straws or loops supplied in the RDT kits were replaced by a transfer pipette (Finnpipette, Helsinki, Finland). Readings were carried out at daylight assisted by a standard electric bulb by two subsequent blinded observers, and test line intensities were recorded [19]. RDT kits had been stored between 18°C and 24°C before use. Laboratory diagnosis of malaria at ITM is accredited in accordance with the requirements of the standard NBN EN ISO 15189:2007.

The questionnaire and the instructions were drafted in Dutch. They were translated into French (both French and Dutch are national languages in Belgium) by a native French speaking professional and both versions were again checked by a professional not involved in the study preparations. Comments and feedback to the questionnaire and instructions raised during the try-out session were addressed during a next IPH referee committee meeting and a final version of the questionnaire was approved.

### Data analysis

Participants were asked to state the RDT brand they used and to report their results in terms of observations (*i.e*. the presence of control and test lines) and interpretations. Interpretations referred to the final diagnosis: participants were offered a free-text option and were invited to submit their answer formulated as a report to the clinician. Participants submitted the results on-line via the IPH-EQA website or sent them by post mail to IPH. The results were converted (answers through the website) or encoded (forms sent by post mail) in an Excel^® ^database (Microsoft Corporation, Redmond, Washington USA).

For analysis, the results of the RDT tests, and answers to the questionnaire were reviewed and grouped. As the intended denominator consisted of the number of participants, only the first RDT was considered in case a participant used more than one RDT brand. Primarily, the interpretations of RDT results (report to the clinician) were considered. In case of an unexpected or incorrect result, the observations (results of control and test lines) were retrieved in order to distinguish between interpretative, analytical and clerical errors. In case a final result in terms of interpretations was not clearly stated (*e.g. *phrasings such as "result depends on microscopy"), it was not included for analysis. For interpretation and scoring of the RDT results, major, minor and very minor errors were defined based on relevance and impact on patient care, they are as listed in Table 3. Continuous variables were assessed for significance using the Student's t-test.

**Table 3:** Score for EQA test results, considered as "report to the clinician".

Correct	• Correct diagnosis and correct report.
**Very minor error**	• Not diagnosing or reporting the possibility of a mixed infection, with non-*falciparum *species as the disregarded species.

**Minor error**	• Missing the diagnosis of non-*falciparum *species.
	• Reporting "positive" when information on confirmation/ruling out of *P. falciparum *is available.
	• Reporting *P. vivax *in stead of non-*falciparum *species.
	• Correct result but with incorrect comment.

**Major error**	• Invalid RDT test result not recognized.
	• Diagnosis of *P. falciparum *missed.
	• *P. falciparum *diagnosed or reported as non-*falciparum *species.
	• Non*-falciparum *species diagnosed or reported as *P. falciparum.*
	• Negative sample diagnosed or reported as "positive".

### Additional analyses

In an attempt to explain for some RDT reporting errors, the package inserts of the RDT kits were reviewed for the "interpretation" section.

To have an idea about the expected parasite densities of malaria samples in the present non-endemic setting, the parasite densities of the samples processed at ITM were retrieved. These samples were obtained from patients attending the outpatient clinic at ITM or sent by Belgian laboratories to ITM for confirmation in the scope of the national reference function. Only the first sample per patient was considered, for the period from January 2000 to June 2010.

## Results

### EQA sessions

The try-out EQA session was performed on June 24^th^, 2009, during summer season. Eight out of nine temperature monitored packages arrived at ITM. The median duration of transport of the samples was 52 hours (range 49 - 144 hours). Mean and maximum temperatures during shipment were 23.6°C (ranges 22.6°C - 25.2°C) and 28.7°C (25.9°C - 32.1°C). The temperatures during shipment had exceeded 25.0°C for a median duration of 630 minutes (range 350 - 6,380 minutes). The formal EQA session was organized on February 22^nd ^2010, during winter season.

Results of the try-out testing were identical to those obtained by RDT testing of fresh samples for the three shipped samples and all the tested RDT brands. The results of Cypress and Ultimed (performed on stored samples at the time of the formal EQA session) were in line with the expected results (HRP-2 and pan-pLDH test lines visible in sample 1, no test lines visible in sample 2, and pan-pLDH line visible in sample 3).

### An overview of participants and malaria RDTs used

The total number of laboratories subscribing to the Belgian EQA "Microbiology" in 2009 - 2010 was 183. A total of 128 subscribed to the session on malaria RDTs. When surveyed, 50 of the 55 non-subscribing laboratories declared not to perform RDTs as part of malaria diagnosis. In other words, 133/183 (72.7%) of clinical laboratories offering malaria diagnosis were using RDTs at the time of EQA, and 128 (96.2%) of them participated to the present EQA session on malaria RDTs.

Table 2 lists the different RDT brands used by the 128 participants, matched with their format and target antigens. Two participants used more than one RDT brand: one of them used two additional brands (SD Bioline and a *P. vivax*-pLDH specific RDT), and another participant used Palutop as part of an internal evaluation procedure. The results of these additional RDTs were not considered for analysis.

### RDT results for the samples

Table 4 displays the results for sample 1 (*P. falciparum*, parasite density of 53,024/μl). The expected result was: "*P. falciparum*, a mixed infection with *P. vivax, Plasmodium ovale *or *Plasmodium malariae *(or in case of the four-band Palutop: "*P. ovale *or *P. malariae*") cannot be excluded". Four participants did not give a final result in terms of interpretation and report to the clinician. Their observations of test lines were correct, but they were not included for analysis. The final denominator consisted of 124 participants. Only a single major error was observed: one sample was reported as negative. However, the observations of test lines reported for this sample were correct (presence of HRP-2 and pan-pLDH lines), suggesting an administrative error. Less than half of the participants scored this sample correct. One third committed the very minor error of not mentioning the possibility of a mixed infection with non-*falciparum *species. The reporting of only 'positive' without mentioning the presence of *P. falciparum *was observed in 16.9% of reports and was considered as a minor error.

**Table 4:** Results for sample 1: *P. falciparum* sample. Eligible answers of 124 participants were included.

	RDT brand						
	
Reported result	Binax	Palutop	Optimal	CareStart	SD Bioline	Ultimed/Cypress	Total (%)
Negative*		1					1 (0.8)
Positive^†^	13	1	2	1	3	1	21 (16.9)
*P. falciparum*^‡^	1	22	10	7	4		44 (35.5)

*P. falciparum *or mixed infection	37	2	11	4	4		58 (46.8)

*major, † minor and ‡ very minor errors, see definitions in Table 3.

Table 5 shows the result for sample 2 (no *Plasmodium *detected). The expected result was "negative" or "no *Plasmodium *antigen detected". Three participants did not answer a final result in terms of interpretation and report to the clinician. Their observations of test lines were correct, but they were not included for analysis. The final denominator consisted of 125 participants. Three major errors occurred. One participant reported "*P. falciparum *or mixed infection". The corresponding observations of the test line results were supporting this result, *i.e. *strong line intensities for both Pf-pLDH and pan-pLDH lines. Two other participants reported the absence of the control line for this sample but failed to report the test result as invalid. Twenty-four participants added a comment to the report: most comments were valuable adjuncts pointing to the need of repeating the RDT (and microscopy) in case of a negative test result and a persistent suspicion of malaria. Four participants added comments that were considered as not correct. One of these comments was raised by two participants, it stated - in case of negative result and persistent suspicion - "to repeat the RDT at the next peak of fever".

**Table 5:** Results for sample 2: *Plasmodium* negative sample. Eligible answers of 125 participants were included.

	RDT brand						
	
Reported result	Binax	Palutop	Optimal	CareStart	SD Bioline	Ultimed/Cypress	Total (%)
*P. falciparum* or mixed infection*			1				1 (0.8)
Absence of control line not reported as invalid*			1		1		2 (1.6)
Negative + comment which is NOT correct†	1	1	1	1			4 (3.2)
Negative + Correct comment	5	4	2	3	5	1	20 (16.0)

Negative	45	21	18	8	5	1	98 (78.4)

*major and † minor errors, see definitions in Table 3.

Table 6 displays the results of the third sample (*P. vivax*, parasite density 3,251/μl). The expected result was "*P. vivax, P. ovale *or *P. malariae*" or "non-*falciparum *malaria". In case of the four-band Palutop the correct result was: *"P. vivax*, mixed infection with *P. ovale *and *P. malariae *not excluded". Three participants did not answer a final result in terms of interpretation and report to the clinician. Their observations of test lines were correct, but they were not included for analysis. The final denominator consisted of 125 participants. None of the participants using Binax observed a test line and therefore all of them missed the expected diagnosis. Minor errors observed were the reports of simply "positive" or "pan-species" (n = 13, 10.5%), without mentioning the absence of *P. falciparum *infection. The report of "*P. vivax*" in case of Palutop was considered a very minor error since the possibility of mixed infection was not mentioned. In case of CareStart, the answer "*P. vivax*" was of note since this brand does not include a *P. vivax*-specific test line.

**Table 6:** Results for sample 3: *P. vivax* sample. Eligible answers of 124 participants were included.

	RDT brand						
	
**Reported result **Reported result	Binax	Palutop	Optimal	CareStart	SD Bioline	Ultimed/Cypress	Total (%)
Negative^†^	51						51 (41.1)
Positive^†^			1	1	2	1	5 (4.0)
Pan-species^†^		1	2	2	3		8 (6.5)
*P. vivax*		23^‡^		5^†^			28 (22.6)
*P. vivax; P. ovale *and *P. malariae *not excluded		2					2 (1.6)

Pan-species, not *P. falciparum*			20	4	6		30 (24.2)

*major, † minor and ‡ very minor errors, see definitions in Table 3.

### Results of the questionnaire

Table 7 lists the numbers of requests for malaria diagnosis processed by each laboratory in 2009, matched to the number of laboratory staff. In line with the low number of requests were the low numbers of RDT tests performed in 2009: 56.8% (71/125) of participants declared to have processed 20 tests or less, another third (44/125, 35.2%) mentioned between 20 and 100 RDTs. More than three quarters of participants (97/120, 80.8%) replied that RDTs had improved the diagnosis of malaria in their setting.

**Table 7:** Cross tabulation of the numbers of laboratory staff involved in malaria diagnosis versus the numbers of requests for malaria diagnosis in 2009.

	Numbers of laboratory staff performing malaria diagnosis				
	
Numbers of requests for malaria diagnosis in 2009	0-5	6-10	11-20	>20	Total
0-10	4	8	16	3	31
11-20	11	7	11	1	30
21-100	4	16	17	12	49
101-500	4	4	5	1	14
>500		1			1
Total	23	36	49	17	125

Table 8 lists the ease of use for each of the RDT brands expressed on a scale from 0 to 10. Although the median scores did not differ much and differences did not reach statistical significance, there was a wider range with a tendency to lower scores for Binax and, to a lesser extent, OptiMAL.

**Table 8:** Ease of use of the different malaria RDTs expressed as a score.

RDT brand	Numbers of laboratories using this brand	Median Score	Range
Binax	52	8	2-10
OptiMAL	23	8	6-9
Palutop	25	9	7-10
CareStart	12	9	8-10
SD Bioline	11	9	8-10

Tables 9 and 10 list the diagnostic strategies during and outside opening hours (weekend and night shifts). A total of 125 participants gave eligible answers on their diagnostic strategy during opening hours, among them there were 113 participants who also offered malaria diagnosis outside opening hours. Of note, five participants noted that they do not perform RDTs on follow-up samples. The vast majority (95.2%) of participants used RDTs as a complement or adjunct to microscopy during opening hours, but only 62.8% did so outside opening hours. Moreover, outside opening hours, 31.1% of them relied on RDTs as the primary (4.4%) or the single tool (25.7%) for malaria diagnosis. In a minority (approximately 5%) of laboratories, the decision of performing a RDT either alone or in conjunction with microscopy was left to the attending clinician.

**Table 9:** Strategy of malaria diagnosis during opening hours as reported by 125 participants.

Diagnostic strategy of malaria during opening hours	Numbers of participants (%)
Microscopy + always RDT	99 (79.2)
Microscopy + RDT for confirmation	18 (14.4)
Microscopy + RDT if requested by the clinician	2 (1.6)
Microscopy and/or RDT, depending on the request by the clinician	3 (2.4)
RDT, if positive or in case of doubt: + microscopy	3 (2.4)

**Table 10:** Strategy of malaria diagnosis outside opening hours as reported by 113 participants.

Diagnostic strategy of malaria outside opening hours	Numbers of participants (%)
Microscopy + always RDT	63 (55.8)
Microscopy + RDT for confirmation	8 (7.1)
Microscopy + RDT if requested by the clinician	2 (1.8)
Microscopy alone	2 (1.8)
Microscopy and/or RDT, depending on the request by the clinician	3 (2.7)
RDT: if RDT positive, microscopy is done instantly; if RDT is negative, microscopy is done the next day	5 (4.4)
RDT + microscopy next day	1 (0.9)
RDT: if positive or in case of doubt: + microscopy	16 (14.2)
RDT alone, no microscopy	13 (11.5)

### Additional analyses

To generate an idea about the parasite densities of patients presenting in Belgium and in the Grand Duchy of Luxembourg, archived data were reviewed at ITM. Table 11 lists the parasite densities of the samples submitted to ITM for each species, for a 10-year period (January 2000 - June 2010).

**Table 11:** Distribution of parasite densities (asexual parasites/μl) per species for the 1066 *Plasmodium* positive samples submitted to ITM for the period January 2000 - June 2010. (Only the first sample per patient was included).

Parasite density	Single infection, species				**Mixed infection**^**†**^
		
Numbers	*P. falciparum*	*P. vivax*	*P. ovale*	*P. malariae*	
0-100	81	3	12	3	0
101-500	93	14	22	3	2
501-5,000	221	52	38	20	4
5,001-250,000	360	59	14	6	5
>250,000	53	1			
Total.	808	129	86	32	11
**Cumulative (%)**					
					
≤100	10.0	2.3	14.0	9.4	0
>100	90.0	97.7	86.0	90.6	100
>500	78.5	86.8	60.5	81.3	81.8
>5.000	51.1	46.5	16.3	18.8	45.5
>250.000	6.6	0.01	0	0	0

^**† **^Mixed infections included *P. falciparum *infection with *P. ovale *(n = 4) or *P. malariae *(n = 5) and *P. malariae *infection with *P. ovale *(n = 1) or *P. vivax *(n = 1).

**Figure 1:**
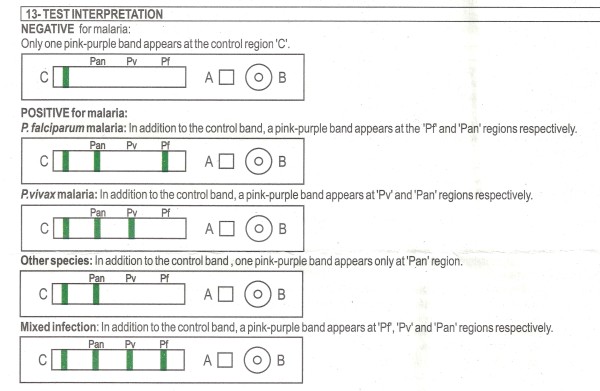
**Package insert of Palutop, test interpretation**. The instructions do not mention the possibility of a mixed infection in case of "*P. falciparum *malaria" and "*P. vivax *malaria".

**Figure 2:**
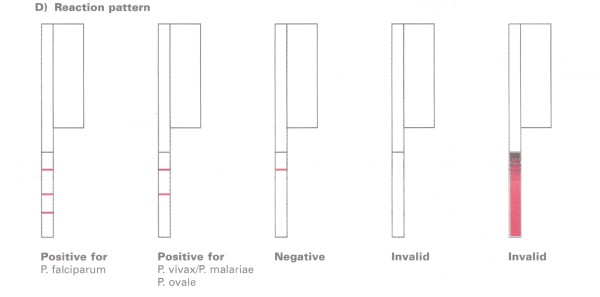
**Package insert of OptiMAL, test interpretation**. The instructions do not mention the possibility of a mixed infection in case of "Positive for *P. falciparum*".

The frequency of not reporting the possibility of a mixed infection in the case of *P. falciparum *(sample 1, 35.3%) and *P. vivax *(22.6%, sample 3) was striking. In sample 1, half of these errors occurred with Palutop and represented the vast majority (22/26) of reports obtained with this brand. For this reason, the package insert of this kit was analysed: for the combination of the "Pf" (HRP-2) and the "Pan" (pan-pLDH) lines, Palutop instructions mentioned "*P. falciparum*" without adding the possibility of a mixed infection with *P. ovale *or *P. malariae*. A similar omission was noted for the *P. vivax *interpretation: the possibility of a co-infection with *P. malariae *and *P. ovale *was not mentioned (Figure 1). OptiMAL kit's instructions neither mentioned the possibility of a mixed infection in the case of the diagnosis "*P. falciparum*" (Figure 2).

**Figure 3:**
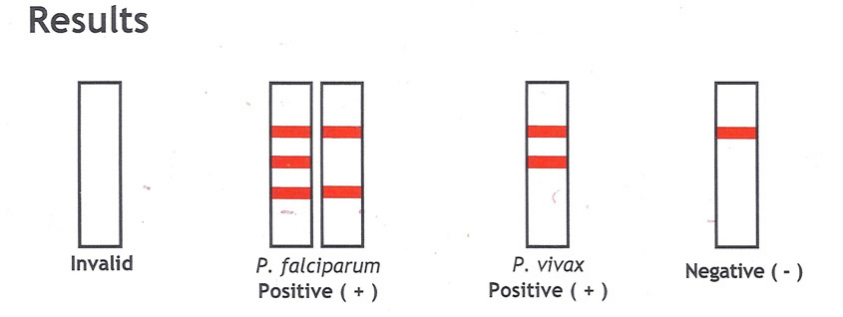
**Package insert of CareStart "Single Kit"**. Test interpretation stating "*P. vivax*" instead of "non-*falciparum *species" when a single pan-pLDH line is visible.

Likewise, there were errors in the interpretation of test lines in the CareStart package insert. From this brand, two versions were available: a version with individually wrapped tests ("Single Kits", "Lab in a pack") and a regular laboratory kit. For the interpretation of a single pan-pLDH line, the former mentioned "pan-species" and the latter "*P. vivax"*, thereby explaining for the unexpected reporting of "*P. vivax*" by five participants (Figure 3, Table 6).

## Discussion

The present study reports the findings of an EQA session on malaria RDTs in a non-endemic setting. Major analytical errors were rare. Minor errors occurred frequently and were related to interpretation or reporting of test results (*e.g. *reporting "RDT positive" without species identification when this was possible based on RDT test line result). Errors in interpretations (*e.g. *the possibility of mixed infections and the identification of the non-*falciparum *species) were embedded in the package insert instructions of some RDTs. Nearly half (48.8%) of participants received ≤20 requests for malaria diagnosis in 2009. During opening hours, 93.6% of participants used RDTs as an adjunct to microscopy whereas outside opening hours, nearly one third of them relied on RDTs as primary or single tool for malaria diagnosis.

As far as known, this is the first report of an EQA on malaria RDTs. It should be noted that the present EQA session suffered from the weaknesses inherent to the EQA method. For instance, only a few samples were offered, precluding comparison of diagnostic characteristics of the different RDT brands. Furthermore, participants were alerted to the importance of the samples and probably might have devoted more attention to them than to routinely submitted samples. Finally, a single EQA session such as the present one will only offer a cross sectional idea about the performances of the participants. On the other hand, the method of EQA has its strengths: it provides an educational stimulus to the participants, allowing them to boost their self-confidence and to show their credibility. Furthermore, EQA sessions including the present one also provide information to the health authorities: they provide insights into the participants' performance levels, may trace problems in test kits and methods and they can survey the use of diagnostic algorithms [21]. A particular strength of the present EQA was its representativeness: in Belgium, subscription to EQA sessions is mandatory for clinical laboratories and the registration fee covers all sessions. This particular condition explains for the high coverage rate (96.2% of all laboratories performing RDTs) of this EQA session.

The try-out EQA session validated the use of clinical samples after storage and shipment. According to WHO findings, antigen stability in sample panels is preserved during storage at -70°C for 20 months; in addition, storage at 4°C before freezing samples does not impair panel quality nor does storage at room temperature for up to 60 days [22]. In light of these findings and the present results, extension of EQA sessions to other settings such as endemic field settings might be feasible. The only difference with fresh clinical samples is the hemolysis caused by freezing: the additional hemolysis however does not interfere with RDT processing but contributes to swift clearance of the background on the nitrocellulose strip [23].

In the present EQA session, analytical errors were rare. Two major errors were observed. The single missed diagnosis of *P. falciparum *(sample 1) was related to an administrative rather than to an analytical error; the unexpected single "*P. falciparum*" report for one out of 26 Palutop tests (sample 2) might also be related to a clerical error (mixing up specimens or laboratory forms) although the reported test line results were in line with the correct result. More of concern was the missed interpretation of invalid test results by two participants, which was considered as a major error: in the absence of a visible control line, test results are not reliable and the test should be repeated. Invalid test results occur rarely (<0.5% of samples tested) but consistently [24-27] and laboratory staff should be alert to this phenomenon.

Interpretation or reporting of RDT results was more subject to errors: simply reporting "positive" without mentioning the species in the case of *P. falciparum *(Sample 1, 16.9%) and non-*falciparum *(Sample 3, 4.0%) or reporting "pan-species" without mentioning that *P. falciparum *was not involved (Sample 3, 6.5%) was considered as a minor error: all relevant information from the RDT results, in particular the presence or absence of the potentially fatal *P. falciparum*, should be exploited.

Sample 2 did not contain *Plasmodium *antigen. Although this was a confirmed malaria-negative sample correctly identified by 97.6% of participants, it should be noted that false-negative RDT results may occur. Reasons for false-negative RDT results in a non-endemic setting are low parasite densities, polymorphisms in HRP-2 and the prozone effect. Low parasite densities are by far the most frequent cause of false-negative RDTs: for *P. falciparum*, they occur more frequently below the threshold of 100 parasites/μl, but at this density non-immune travelers can present with symptoms [19,26-29]. From Table 11 can be read that *P. falciparum *samples with parasite densities below 100/μl can be expected in about 10% of samples. In such cases - and as raised by some participants as a comment to their report of sample 2 - repeated testing after 8 - 12 hours is advised, for up to four consecutive samplings [30,31]. Of note, the comment of awaiting a next peak of fever for repeating the RDT was considered as a minor error: although there are variations in antigen production during the cycle, there is no clear relation between the concentration of antigens and any particular moment of the cycle, yet the peak of fever [29] and a periodic fever pattern does not occur in most of the *P. falciparum *infections. Polymorphisms in HRP-2 may give rise to variations that are less likely to be picked up by current RDTs; they are geographically confined to the Asia-Pacific and South-American regions [32-34]. For the prozone effect, high antigen concentrations block all available binding sites of both the detection and the capture antibodies, thereby hindering test line generation. It occurs at high parasite densities of *P. falciparum *and presents mostly as test lines with spuriously low intensity, although complete negative results do occur. It tends to occur in HRP-2 based but not in Pf-pLDH based tests [35].

The failure of Binax to detect *P. vivax *in sample 3 was not considered as a major error in light of the ability of RDTs to detect non-*falciparum *species. In addition, EQA sessions are not designed as a side-to-side comparison of different brands nor as an evaluation of RDT test characteristics. However, it demonstrates the lower sensitivity of RDTs to detect non-*falciparum *species as compared to the detection of *P. falciparum *[19,26-29,36]. For Binax (in its most recent generation), compiled sensitivity for the diagnosis of *P. vivax *has been calculated to be 68.9% [29]. In a population of returned travelers, Binax displayed sensitivities of 86.7% for pure *P. vivax *samples [30]. In comparison, OptiMAL which was run side-to-side in the latter study displayed a higher sensitivity for *P. vivax *(91.0%), at the expense of a lower sensitivity for *P. falciparum*. Challenged to a panel of stored samples at ITM, SD Bioline, Palutop and CareStart showed overall sensitivities for the detection of *P. vivax *of 87.5%, 66.0% and 77.6% respectively [19,26,28], with, as for Binax in the current sample, false-negatives not limited to low parasite densities. For *P. ovale *and *P. malariae*, reported sensitivities have been even lower [19,26,28,36]. As for *P. falciparum*, the sensitivity for the non-*falciparum *species is related to parasite density, with a threshold value at 500/μl [19,24-26,28] or even at 5,000/μl [12] but false negatives also occur above these densities. Parasite densities below these 500 and 5,000 threshold values occur in 23.0% and 67.6% of the non-*falciparum *samples in the Belgian reference laboratory for malaria (Table 11).

Not reporting the possibility of a mixed infection of *P. falciparum *with non-*falciparum *species (Sample 1, 35.5%) was considered a minor error, as this has no impact on the short-term patient care: in case of an additional *P. vivax *or *P. ovale *species, the persistent liver schizonts have to be eradicated with primaquine treatment, but there is time to await this information from the reference laboratory. However, it was striking that this error as well as the error of not reporting the possibility of mixed infections in the case of *P. vivax *(sample 3, 22.6%) were embedded in the package insert instructions of Palutop and OptiMAL. Likewise there were errors on interpretation in the Carestart package insert instructions, with incorrect labeling of the test lines in both versions. Manufacturers should be encouraged to revise and adapt their instructions where needed, especially with regard to test line interpretations.

The questionnaire confirmed the low critical volume in terms of requests for malaria diagnosis: assuming a 5-10% positivity rate for patients suspected of malaria, it is clear that laboratory technicians have little exposure to malaria-positive slides. Even in those laboratories with high numbers of requests, the number of performances per technician will be reduced by a high number of laboratory staff participating in the diagnosis. This is comparable to the situation in other countries, with about half of laboratories in the U.K. carrying out less than 100 malaria requests each year and about 10% of laboratories fewer than 10 [18]. In France, about 60% of 3,300 surveyed laboratories declared to have seen a malaria case the previous year, and only 5% made the diagnosis of malaria five times or more yearly [37].

The number of participants (80.8%) replying that malaria RDTs had improved the diagnosis of malaria in their setting was much higher than revealed by a survey in the UK (12.6% out of 305 respondents stated that RDTs had revised their malaria diagnosis) [18]. Although the meaning and phrasing of the latter question were different ("revised" versus "improved"), the difference in numbers is striking. It may be attributed to a growing experience with malaria RDTs in clinical laboratories.

The wider range with a tendency to lower scores for ease of use for Binax and, to a lesser extent, OptiMAL may be explained by the fact that these kits include more steps than the more recently released one-step kits such as CareStart and SD Bioline. In ITM experience as well as that of others [7,38], particular problems can arise with the use of the transfer device: without having processed high numbers of samples, it may be difficult to master the RDT kit's loops, straws or capillary tubes, which are frequently small and not user-friendly. The volume of blood used to run the test is critical: an excess of blood may increase the risk and/or the intensity of prozone effect or may mask a faint line due to a bad clearance of the strip, while a shortage of blood may decrease the sensitivity of the test.

Of particular interest are the diagnostic strategies during and outside opening hours. Whereas the vast majority of participants used RDTs as a complement or adjunct to microscopy during opening hours, the reliance on RDTs as the primary or the single diagnostic tool during night and weekend shifts is of concern. In the UK-survey of 2006 a similar tendency was observed: less than 5% of 327 surveyed laboratories used exclusively a RDT during opening hours, versus 15-20% outside opening hours [18]. The extent of the potential risks as a result of this strategy may be serious especially since, according to a survey in Portugal, about half of the requests for the diagnosis of malaria arrive outside opening hours, accounting for 60% of the diagnoses [39]. In view of their strengths, RDTs are a valuable adjunct for malaria diagnosis and should - in the authors' opinion - be used together with microscopy in all cases of malaria suspicion in non-endemic settings. Conversely, RDT limitations do not justify them as the unique tool for diagnosis in non-endemic settings: microscopy is needed to recover diagnosis that may be missed by RDTs (prozone effect, non-*falciparum *species and low parasite densities), and also to assess signs of severity (elevated parasite densities and the presence of schizonts and haemozoin in the case of *P. falciparum*). It is important to ascertain reliable diagnosis of malaria during and outside office hours, and competent microscopy should be in reach at all times. To minimize the risk of microscopy errors and as an in-service training and feedback, all positive and doubtful samples should be submitted to the malaria reference laboratory.

The policy of leaving the decision on the choice of RDT versus microscopy to the attending clinician may result in not performing the RDT (thereby not exploiting possible information generated by the RDT) or not performing microscopy. This policy is probably related to the reimbursement system of medical costs in Belgium: national health insurance reimburses only laboratory analyses that are explicitly requested by the clinician. Hospital-based diagnostic and treatment algorithms can guide the choice and priorities of laboratory tests, but for the individual patient however, the ultimate request of RDT, microscopy or both depends on the clinician's decision.

The additional comment of five participants that they do not perform RDTs on follow-up samples is a correct policy. Indeed, HRP-2 may persist in the circulation for up to several weeks. Aldolase and pLDH depend on the living parasites and they rapidly decline after start of correct treatment, but their use is limited because they are also expressed by gametocytes [40]. Consequently, monitoring of treatment efficacy should be done by microscopy only.

## Conclusion

From the present EQA session, it is clear that RDTs are an essential part of malaria diagnosis in most diagnostic laboratories in Belgium and the Grand Duchy of Luxembourg. According to the results of the present EQA, it can be concluded that analytical errors in the performance of RDTs are rare. Errors are mainly related to the interpretation and reporting of RDT results, partly due to errors in the package insert. Laboratory staff has limited exposure to malaria positive samples in this non-endemic setting. Whereas during opening hours, RDTs are used as a complement or adjunct to microscopy, there are about one third of participants that rely on RDTs as the primary or the single diagnostic tool outside opening hours, which should be avoided.

## List of abbreviations

EDTA: Ethylene diamine tetra-acetic acid; EQA: External quality assessment; FIND: Foundation for Innovative New Diagnostics; IPH: Belgian Scientific Institute of Public Health; HRP-2: Histidine-rich protein 2; INRB: Institut National de Recherche Biomédicale; ITM: Institute of Tropical Medicine; *P: Plasmodium*; Pan-pLDH: pan *Plasmodium*-specific parasite lactate dehydrogenase; PCR: Polymerase chain reaction; Pf-pLDH: *Plasmodium falciparum-*specific parasite lactate dehydrogenase; pLDH: parasite lactate dehydrogenase; Pv-pLDH: *Plasmodium vivax*-specific parasite lactate dehydrogenase; RDT(s): Rapid diagnostic test(s); WHO: World Health Organization.

## Competing interests

The authors declare that they have no competing interests.

## Authors' contributions

PG, KV, MvE and JJ designed the external quality assessment. PG, MvE, and JJ carried out the try-out EQA session. KV and MvE carried out the formal EQA session. PG, PM, KV and JJ analysed and interpreted the results. All authors drafted and critically reviewed the manuscript and approved the final manuscript.
